# Revisiting *Trigonella foenum-graecum* L.: Pharmacology and Therapeutic Potentialities

**DOI:** 10.3390/plants11111450

**Published:** 2022-05-29

**Authors:** Theysshana Visuvanathan, Leslie Thian Lung Than, Johnson Stanslas, Shu Yih Chew, Shalini Vellasamy

**Affiliations:** 1 Department of Medical Microbiology, Faculty of Medicine and Health Sciences, University Putra Malaysia, Serdang 43400, Malaysia; theyshu_89@hotmail.com (T.V.); leslie@upm.edu.my (L.T.L.T.); chewshuyih@upm.edu.my (S.Y.C.); 2 Department of Microbiology and Parasitology, School of Medicine, Faculty of Medicine, Bioscience and Nursing, MAHSA University, Jenjarom 42610, Malaysia; 3 Department of Medicine, Faculty of Medicine and Health Sciences, University Putra Malaysia, Serdang 43400, Malaysia; rcxjs@upm.edu.my

**Keywords:** alkaloids, fenugreek, pharmacological potential, phytochemicals, *trigonella foenum-graecum*, saponins

## Abstract

Fenugreek (*Trigonella foenum-graecum* L.) is a medicinal plant that has been used as a food condiment as well as for its multiple therapeutic characteristics since ancient times. Fenugreek plant grows up to 60 cm in height, and its seeds are golden-yellow rhomboidal-shaped. Though fenugreek is more commonly known for its seeds, the leaves and stem have also been reported to have medicinal uses. These properties exhibited are due to the content of the secondary metabolites, also known as phytochemicals, in the fenugreek plant. Such metabolites are alkaloids, saponins, tannins, phenols, and many others. Fenugreek has been used traditionally for numerous indications, such as aid in labour, lactation stimulant, and laxatives. In modern research, there have been several animal and clinical studies that have shown therapeutic effects of fenugreek when taken orally. Fenugreek is a suitable plant candidate with a high prospect of being used as a credible medicinal plant to derive new drugs. This review aims to summarize the physical and chemical properties of fenugreek and its bioactive compounds that have been isolated for medicinal purposes and discusses the traditional and pharmacological uses of fenugreek.

## 1. Introduction

Despite the incredible advances in medicine development, herbal crops are still widely used for treating and preventing a variety of diseases due to their medicinal and nutraceutical characteristics. *Trigonella foenum-graecum* L., or also commonly known as fenugreek, is known to be one of the plants with these traits. It is from the family of Fabaceae and is a self-pollinating annual herbaceous aromatic crop, also known as bird’s foot, Greek hayseed, halba, and methi [1]. Its origin is India and Northern Africa; however, it is now widely cultivated in Northern Africa, Europe, South Asia, Argentina, and Australia. Fenugreek is mainly produced in India, which accounts for 80% of the total world production [2]. Fenugreek seeds and leaves are used as a spice and ingredient in culinary preparation in several countries. It is used as a functional and traditional food, as well as in nutraceuticals and physiological application. Because of its high fibre, protein, and gum content, fenugreek has recently been utilized as a food stabilizer and emulsifying agent.

Fenugreek is known to be one of the world’s most ancient medicinal herbs, in relation to which the seeds and leaves are used as a treatment in various ailments [3]. The leaves and seeds of *T. foenum-graecum* are extensively utilized to make extracts and powder for therapeutic applications in numerous investigations. There have been several preliminary animal and human trials that demonstrated fenugreek exhibiting hypoglycaemic, hypolipidemic, and hypocholesterolemic effects. *T. foenum-graecum* has also been reported to possess anti-fertility, anticancer, anti-parasitic, and antimicrobial effects [4]. Fenugreek belongs to the *Fabaceae* family, *Trigonella* genus, and *Foenum-graecum* species.

## 2. Physical and Chemical Properties of *T. foenum-graecum*

Fenugreek (*T. foenum-graecum*) seeds sprout in three days once sown in suitable soil. The seedlings grow erect or semi-erect up to 30 to 60 cm of height [5]. It is a self-pollinating annual leguminous bean that aids in soil nourishment and nitrogen fixation. Fenugreek plants are green in colour and slender in shape, with yellow–brown pods bearing 10 to 20 seeds. Fenugreek seeds are brownish-yellow in colour, small, rhomboidal-shaped, and hard [5] seeds are 3 to 6 mm long, 2 to 5 mm wide and 2 mm thick [3]. Crude fenugreek seeds are known for their pleasantly bitter and maple flavour. They are glutinous, fibrous, and sticky in texture and biologically endospermic in nature. A hard centre and yellow embryo is surrounded by a corneous wider layer of white and translucent endosperm in fenugreek seeds [6]. A diosgenin component has been found to be present in the embryo.

Fenugreek seeds consist of 45 to 60% carbohydrates, in which mucilaginous fibre (galactomannans), 20 to 30% proteins high in tryptophan and lysine, 5 to 10% fixed oils (lipids), pyridine alkaloids, mainly choline (0.5%), trigonelline (0.2–0.38%), gentianine, and carpaine, the flavonoids apigenin, orientin, luteolin, quercetin, vitexin, and isovitexin, free amino acids, such as 4-hydroxyisoleucine (0.09%), arginine, lysine, and histidine calcium and iron, saponins (0.6–1.7%), glycosides yielding steroidal sapogenins on hydrolysis (diosgenin, yamogenin, tigogenin, neotigogenin), cholesterol and sitosterol, vitamins B, A, C, and nicotinic acid, and 0.015% volatile oils (*n*-alkanes and sesquiterpenes) [6,7].

Fenugreek seed is known to consist of fibre, gum, and various chemical constituents and volatile contents. At an alkaline pH, the protein in fenugreek is more soluble [3]. Fenugreek has a protein content of 23 to 26%, a lipid content of 6 to 7%, and a carbohydrate content of 58%, of which roughly 25% is dietary fibre [6]. Furthermore, fenugreek contains 33 mg of iron per 100 g dry weight, making it a good source of iron. The leaves have an approximate moisture content of 86.1%, 4.4% protein, 0.9% fat, 1.5% minerals, 1.1% fibre, and 6% carbs [6]. Calcium, iron, phosphorus, riboflavin, carotene, thiamine, niacin, and vitamin C are all vitamins and minerals found in the leaves. Fresh fenugreek leaves contain approximately 220.97 mg of ascorbic acid per 100 g of leaves, and approximately 19 mg/100 g of β-carotene is present [6].

The fenugreek seed has been found to contain a number of chemical compounds, commonly known as phytochemicals. They contain various types of alkaloids, flavonoids, and saponins, with the saponins showing the highest concentration with 4.63 g per 10 g [7]. Fenugreek contains about 35% alkaloids, mainly trigonelline. Fenugreek seeds also contain more than 10 mg of flavonoid per gram of seed, as well as a minor amount of volatile and fixed oils. [8]. The oils were rich in linoleic acid (42.71–42.80%), linolenic acid (26.03–26.15%), and oleic acid (14.24–14.40%) [8]. Fenugreek essential oil from the seeds (>5%) is rich in neryl acetate (17.3%), camphor (16.3%), β-Pinene (15.05%), β-caryophyllene (14.63%), and 2,5-dimethylpyrazine (6.14%) [8]. The phytochemical analysis of fenugreek revealed that the majority of flavonoids occur as glycosides, which are intricate and attributed to C-glycosidic and O-glycosidic bonding with carbohydrates. Flavonol glycosides found in fenugreek include quercetin-3-O-rhamnoside (quercitrin), vitexin-7-Oglucoside (afroside), and apigenin-6-C-glucoside (isovitexin). The alkaloid and volatile compound present are the two main chemical constituents that cause the bitter taste and the odour of the seeds. The seeds of fenugreek contain about 0.1 to 0.9% of diosgenin, which is a type of steroidal sapinogen [6]. Polyphenol chemicals, such as rhaponticin and isovitexin, are known to be the primary bioactive compounds in fenugreek seeds.

The bioactive compounds found in the fenugreek have been determined and isolated by researchers using different methods. One of the most common methods of isolation and characterization is chromatography paired with mass spectrophotometry. The high-performance liquid chromatography (HPLC) system was used to identify and quantify bioactive substances, such as trigonelline, isoorientin, orientin, vitexin, and isovitexin. The high-performance liquid chromatography hybrid electrospray quadrupole time-of-flight mass spectrometric (HPLC-ESI-QTOF-MS/MS) approach was used to identify bioactive chemicals much faster [9]. The polyphenol in fenugreek extracts was identified using HPLC combined with negative ion electrospray ionisation mass spectrometry and diode array detection in research [10]. Fenugreek seed oil was extracted, and chemical compositions and bonding were assessed using gas chromatography coupled to mass spectrometry (GC-MS) and Fourier transform infrared spectroscopy (FT-IR) analyses in another study. Linoleic acid, palmitic acid, pinene, 4-Pentyl-1-(4-propylcyclohexyl)-1-cyclohexene, and linoleic acid methyl ester were the most abundant components in the extracted oil [11].

**Figure 1:**
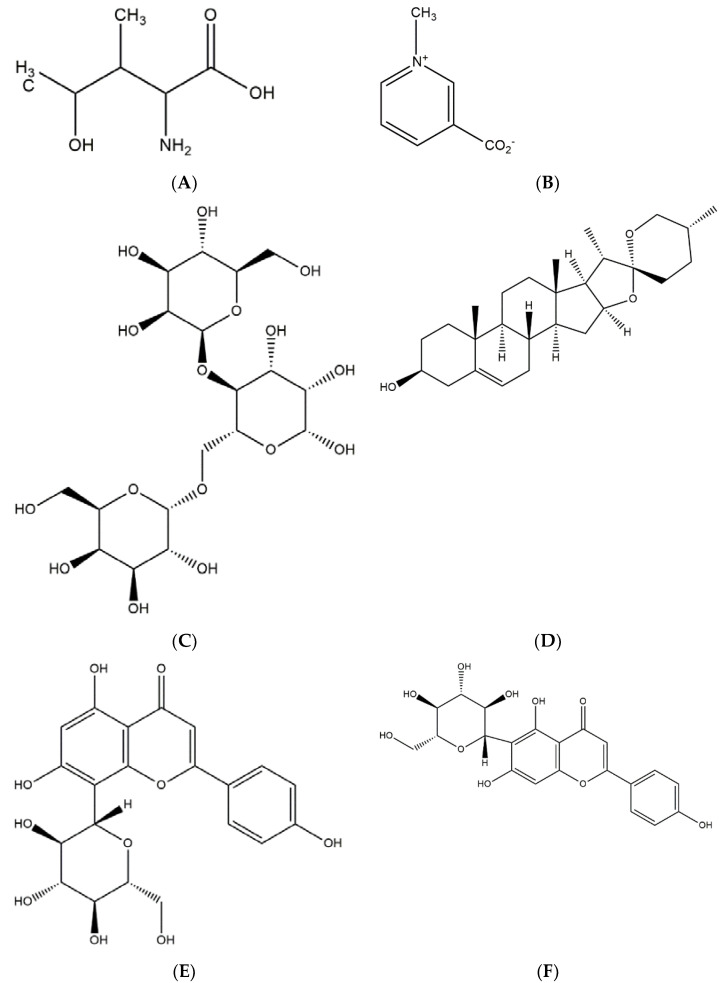
Chemical constituent of fenugreek and its chemical structure and pharmacological effect. (**A**) 4-hydroxyisoleucine, known to have antidiabetic properties. (**B**) Trigonelline, known for its hypoglycaemic activity. (**C**) Galactomannan, a compound with hypoglycaemic effect. (**D**) Diosgenin, associated with dyslipidemia. (**E**) Vitexin, an antioxidant compound. (**F**) Isovitexin, an isomer of vitexin with similar properties.

Trigocaumarin, nicotinic acid, and trigonelline are only a few of the alkaloids found in the plant’s stem. The stem also consists of 28% mucilage, volatile and bitter fixed oil, 22% proteins, and yellow colouring substance. Table 1 shows the vitamin profile of fenugreek, whereas Table 2 summarises the chemical contents found in fenugreek seed, while Figure 1 depicts their chemical structures and pharmacological effects [12,13].

## 3. Traditional Uses of *T. foenum-graecum*

*Trigonella foenum-graecum* has been utilized as a medicinal plant in Central Asia since approximately 4000 BC. The benefits and medicinal purposes have been found reported in one of the oldest medicinal documents, the Ebers papyrus. Traditionally, *T. foenum-graecum* L. has a long history of medical uses in Ayurvedic and Chinese medicine as a demulcent, lactation stimulant, and laxatives [14]. In ancient Rome, fenugreek was used to aid labour, period cramps, and as a tonic for metabolism [15]. Whereas, in ancient Egypt, fenugreek was used to boost milk production in breastfeeding mothers, and modern Egyptian women still consume these seeds to alleviate menstrual cramps. It has also been used as folk medicines to treat cellulitis, boils, and tuberculosis. In the 19th century, fenugreek remained the main ingredient in patent medicine for dysmenorrhoeal and postmenopausal symptoms [6]. Besides that, Yadav and Kaushik [16] mentioned that the gelatinous texture of fenugreek seeds may have some topical effect in soothing irritation caused by eczema. Traditional Chinese medicine also uses fenugreek seeds in kidney problems and kidney stones as fenugreek reduces the amount of calcium oxalate, which is the crystal that contributes to the formation of kidney stones. Fenugreek is also known to help clear congestion and used as a detoxifying agent in removing toxic wastes, dead cells, and trapped protein through the lymphatic system [6]. Table 3 lists the traditional uses of fenugreek throughout time.

## 4. Pharmacological Uses of *T. foenum-graecum*

### 4.1. Hypoglycaemic Effects

Fenugreek is no longer regarded solely as a folk remedy as of recent years. Fenugreek’s therapeutic benefit in treating many health concerns has been demonstrated in numerous in vitro, in vivo, and clinical trials. Fenugreek has been shown to have anti-hyperglycaemic properties in both humans and animals with type I and type II diabetes. However, the precise mechanism of action of fenugreek in producing this effect is still unknown. Fenugreek enhances peripheral glucose utilisation and tolerance in non-insulin-dependent diabetic individuals, according to Raghuram et al. [17]. Clinical studies by Snehlata and Payal [18] show improvement in glycaemic control among patients with mild type II diabetes mellitus. They believe that the galactomannan-rich soluble fraction of fenugreek is responsible for the hypoglycaemic activity because the fibre slows stomach emptying, delaying the uptake of glucose in the small intestine. Dialyzed fenugreek seed extract had hypoglycemic action comparable to insulin, according to a study of alloxan-induced diabetic rats [19]. Gupta, Gupta, & Lal [20] reported the results of a double-blind placebo study among newly diagnosed patients with type II diabetes mellitus. The authors concluded that the use of fenugreek seeds improves glucose control as well as decreasing insulin resistance. A study was conducted by Kannapan & Anuradha [21] in which fructose-feeding rats were treated with fenugreek-seed-derived polyphenols in comparison with metformin, a commercial anti-diabetic drug. It was concluded that fenugreek seed polyphenols improved insulin signalling and sensitivity compared to metformin-treated rats. In an experiment by Hannan et al. [22], type 2 diabetic rats were given oral fenugreek-seed-derived soluble dietary fibre (SDF) for 28 days. A reduction in serum glucose and an increase in liver glycogen was observed, suggesting that the anti-diabetic effect of the SDF was through the inhibition of carbohydrate digestion, absorption, and enhanced peripheral insulin. A rat animal model was recently used to compare the pharmacokinetics of metformin, a first-line anti-diabetic drug, with and without the concomitant administration of fenugreek extract. According to the findings, taking fenugreek and metformin at the same time boosted the metformin bioavailability and reduced the drug distribution volume by 70% [23]. The researcher concluded that this combination could be a useful way to control the blood sugar levels in diabetic patients.

### 4.2. Hypocholesterolemic Effects

Fenugreek has also shown to have a hypocholesterolemic effect, and many studies have reported that it is able to reduce serum cholesterol. Adults with hypercholesterolemia who were administered powder-form germinated fenugreek seeds for a month experienced significant reductions in total cholesterol (TC) and low-density lipoprotein (LDL). Patients with coronary artery disease and type II diabetes who took fenugreek orally saw a reduction in blood lipids, total cholesterol, and triglycerides without impacting high-density lipoprotein [8]. The fibre content of the fenugreek seed is what appears to reduce the cholesterol production rate in the liver. The soluble fibre also decreases the reabsorption of bile acids in the gut, thus increasing the amount of cholesterol and bile acids excreted through defecation. As a result, there is an increase in the need of cholesterol for the biosynthesis of bile acid; thus, the body resorts to using up the blood cholesterol [24]. Diosgenin, the primary saponin compound in fenugreek, has the capacity to inhibit cholesterol absorption, to decrease liver cholesterol concentration. Stark and Madar [25] tested the cholesterol levels of ethanolic fenugreek seed-extract-fed rats. They observed an 18 to 20% reduction in plasma and liver cholesterol. The saponin-like active chemicals in the ethanolic fenugreek extract may have interacted with bile salts and altered the lipid metabolism, according to the authors. Bile acids combine with saponins produced from fenugreek to form micelles that are too big for the gut to digest [16]. Sharma et al. [26] suggested that the reductions in triglyceride (TG) and LDL in fenugreek-treated adults are due to the pectin component that absorbs bile salt. Diabetes mellitus type II is often correlated with dyslipidaemia. The effect of fenugreek seed powder solution on the lipid profile of newly diagnosed type 2 diabetic individuals was investigated. For 30 days, the test subjects were administered 25 mg of fenugreek seed powder, which resulted in significant reductions in TC, TG, and LDL (*p* > 0.001) [27]. Recently, a group of researchers conducted a meta-analysis that showed that fenugreek supplementation has lipid-lowering activity. They analysed a total of fifteen randomised clinical studies and concluded that the trials indicated a significant impact in lowering the total cholesterol, triglyceride, and low-density lipoprotein levels and increasing the high-density lipoprotein (HDL) [28]. Furthermore, they pointed out that there were no significant alterations in the TG, TC, and LDL between the pre- and post-fenugreek studies.

### 4.3. Immunomodulatory

Immunomodulatory effect refers to an agent that promotes or suppresses immunological responses, and fenugreek has been documented to have this effect. Bin-Hafeez et al. [29] studied the immune responses of Swiss albino mice treated with aqueous fenugreek extract at 50, 100, and 200 mg doses. Based on their studies, they observed a stimulatory effect on the body and organ weight, haemagglutinin titre, quantitative haemolysis assay, late-type hypersensitivity response, and plaque-forming assay. There was an increase in the organ weight of the thymus, kidneys, and liver, increase in the delayed-type hypersensitivity, elevated plaque-forming cells response, as well as the phagocytic index and macrophage phagocytic capability both increased significantly. In another study by Tripathi et al. [30], the immunomodulatory effect of the ethanolic extract fenugreek exhibited a significant increase in phagocytic index and antibody titre in normal immune status mice, indicating the stimulation of humoral immunity. In delayed-type hypersensitivity, it also demonstrated a decrease in the mean difference in paw thickness, indicating that the extract modifies anti-inflammatory properties. Researchers conducted an in vitro study to determine the chemical mechanism by which methanolic fenugreek extract exerts its influence on macrophage polarisation [31]. The studies concluded that the extract regulates the expression of the pro-inflammatory marker and immunoregulator marker M1 and M2, respectively, in the THP-1 macrophages cells. They also suggested that this response may be potentially through the NF-κB activity. Rao et al. [32] recently conducted a study on streptozotocin-induced diabetic rats to assess the immunomodulatory potential of fenugreek and *Coccinia indica* Wight et Arn (methi) extracts alone and in combination with glibenclamide, a popular antidiabetic medication. In comparison to the group treated with glibenclamide, which showed no significant changes in immunomodulatory cells, the study found a synergistic impact of the two extracts in raising CD4+ and CD8+ values.

### 4.4. Antimicrobial Activity

For decades, scientists have been studying the antimicrobial properties of various plants in the hope of the development of novel therapeutics, among them being fenugreek. In a study by Haouala et al. [33], aqueous extraction of different parts of fenugreek plant and various solvent extractions of fenugreek plant were completed to determine the action against fungal strains. Dharajiya et al. [14] studied the antibacterial activity of fenugreek extract based on the zone of inhibition against pathogenic bacteria *E. coli*, *P. aeruginosa*, and *B. cereus*. Similar studies were conducted by Chalghoumi et al. [34] and Sharma et al. [26] by testing the antimicrobial activity of the leaves, seeds, and stem extract with different solvents via the well diffusion method. Methanolic and aqueous extracts of fenugreek seed were also evaluated against Gram-positive and Gram-negative bacteria in a study. Based on their testing, they revealed that methanolic extract had an antibacterial affect, but the aqueous extract did not show any activity. In conjunction with all these studies, the antimicrobial potency and severity of effects vary depending on the plant components and microbe species used, as well as the extraction solvent.

### 4.5. Anticancer Activity

Cancer is one of those diseases with a high mortality rate, and researchers are still working on understanding the manifestation of the disease as well as discovering new drug and therapy treatments for the condition. Fenugreek has been reported to have anticarcinogenic potency on cancer models using cancer cell lines and animal models. Shabbeer et al. [35] demonstrated that prostate cancer cell lines, breast cancer cell lines, and pancreatic cancer cell lines are all selectively cytotoxic to fenugreek extract, but normal cell lines are not. However, based on their studies, they concluded that the potent effect is shown in the whole extract compared to a purified compound as the purified compound is not capable of differentiating between cancer and normal cells. A similar study found that fenugreek extract has a selective cytotoxicity impact in vitro against a panel of cancer cell lines, including T cell lymphoma [36]. The existence of anticarcinogenic chemicals gingerol, cedrene, zingerone, vanillin, and eugenol was discovered using a gas chromatography–mass spectrometry (GC-MS) study of the fenugreek extract. Alcoholic fenugreek extract showed in vitro cytotoxicity against IMR-32, a neuroblastoma cell line, and HT-29, a cancer cell line [37]. According to the investigation carried out by Sebastian & Thanpan [38], MCF-7 cells, a breast cancer cell line, showed a decrease in cell viability and early apoptotic changes when treated with ethanolic fenugreek extract. A similar study was conducted to investigate the effects of methanolic fenugreek extract in vitro using breast cancer cells MCF-7 and SK-BR3, followed by an oral acute toxicity study in a Swiss albino mice model [39]. The results showed that the fenugreek extract responds in a dose-dependent manner, with an anti-metastatic effect, induced inhibition of cell migration, and increase in late apoptosis in both cancer cell lines. The data revealed an upregulation of p53, which suggests that the fenugreek effect is associated with the signalling pathway that prevents further DNA mutation and induces cell death. The in vivo acute toxicity data showed that the oral administration of fenugreek extract did not have any toxic effect in mice. Studies were also completed to investigate the anticancer properties of germinated fenugreek seed extract. Such a study was conducted by Almalki & Naguib [40] involving the BXPC-3 pancreatic cancer cell line and albino mice. The data showed that the aqueous germinated fenugreek seed extract was efficient against BXPC-3 cell lines, with an IC50 of 25 g/mL. In the in vivo investigation, the histopathology revealed that the fenugreek-treated group had better pancreatic tissue with very minor lesions than the non-treated group. Furthermore, the treated group of mice showed an increased survival rate.

### 4.6. Antioxidant Property

Oxidative damage of proteins and lipids is caused by the overproduction of reactive oxygen species. These damages are associated with chronic degenerative diseases. There have been several studies completed that suggest fenugreek as a potential antioxidant. Bukhari et al. [41] reported that alcoholic fenugreek extract has a radical scavenging activity. Besides that, another study by Bhatia et al. [42] demonstrated the defensive effect of fenugreek on lipid peroxidation and enzymatic antioxidant on cyclophosphamide-treated mice by evaluating the lipid peroxidation and antioxidants in the mice urine bladder. Khole et al. [13] isolated two flavonoid compounds, vitexin and isovitexin, from germinated fenugreek seed, which were shown to have antioxidant activity. Joglekar et al. [43] studied the antioxidant properties by lowering power, nitroblue tetrazolium chloride (NBT) assay, and hydrogen peroxide (H_2_O_2_) scavenging. Fenugreek showed the greatest superoxide and free radical scavenging. They concluded that the antioxidant activity is associated with the high phenolic content in the fenugreek. A recent study looked at the effects of fenugreek administration on the antioxidant defence systems of ageing mice’s livers. Because of the reactive oxygen species (ROS) present in these cells, aged mammals have higher levels of apoptosis and oxidative stress, primarily in sinusoidal endothelial cells and bile ducts. Tewari et al. [44] conducted a study on 12-month-old mice to evaluate the activities of the endogenous defence mechanisms, such as superoxide dismutase (SOD), glutathione reductase (GR), and glutathione peroxidase (GPx). Elevated SOD reduced GPx and GR were observed in this study, suggesting that fenugreek is linked to a reduction in reactive oxygen species, which results in a feedback regulation loop that lowers GPx and GR levels. Their findings demonstrated that fenugreek had a favourable effect on the control of hepatic enzymes in aged mice when taken combined. Furthermore, Akbari et al. [11] isolated fenugreek seed oil and used GC-MS to assess its chemical components and bonding. A total of 23 compounds were isolated, and the major compounds were linoleic acid (54.13%) and palmitic acid (16.21%). Both 2,2-Diphenyl-1-picrylhydrazyl (DPPH) and 2,2′-azino-bis(3-ethylbenzothiazoline-6-sulfonic acid) (ABTS) assays showed that the extracted fenugreek oil has strong antioxidant radical scavenging activity.

### 4.7. Hormonal Effects

There have been studies suggesting that fenugreek has an influence on the hormonal activity of the body. This could be because of the phytoestrogen present in the fenugreek. Phytoestrogens are herbal compounds with estrogenic activity. There have been several studies conducted investigating the impacts of fenugreek in treating primary dysmenorrhea. In the absence of any underlying pelvic illness, primary dysmenorrhea is characterised by spasmodic abdominal discomfort during menstruation [45]. Younesy et al. [46] investigated the effects of fenugreek seeds on the severity of primary dysmenorrhea in unmarried students in a double-blind, randomised, placebo-controlled experiment. The fenugreek group had a considerably higher pain reduction (*p* < 0.001), and the length of pain decreased between the two subsequent cycles, according to the findings. A similar clinical trial was carried out to determine the efficacy and safety of fenugreek seed on the intensity of pain in patients with primary dysmenorrhea. A 66.89% reduction in lower abdominal pain was observed in the test group, which received oral fenugreek supplements [47]. Menopausal osteoporosis is also another hormone-related disorder in which oestrogen deficiency is directly linked to bone resorption. The current available treatment for this condition is hormone replacement therapy (HRT). A recent study was conducted to evaluate the role of oral fenugreek seed extract on the bone structure and mechanical properties of ovariectomised Wistar rats [48]. The results of this study show that supplementing with fenugreek seed extract enhanced the maximal flexor force required to break the femur. In addition, in ovariectomized rats, the treated group demonstrated improvements in the dry weight of the tibia, bone microstructure, and avoided trabecular bone loss. Overall, the researchers believe that the steroidal phytoestrogens contained in fenugreek may be responsible for its beneficial effect on bone mechanics and strength. There have also been a few studies highlighting the efficacy of fenugreek in enhancing milk production. Sevrin et al. [49] conducted an in vivo investigation using pregnant Sprague–Dawley rats to see if fenugreek boosted the milk supply in a rodent model. Two model groups were tested under different conditions, which are increased in litter size and maternal dietary restriction. The effect of fenugreek on milk production was measured using a deuterium oxide enrichment method. The outcome of the experiment showed that fenugreek produced an increase in milk flow for the test group under appropriate physiological conditions and confronted with increased litter size. In the test group placed under physiological lactation conditions following dietary protein restriction, however, fenugreek proved unsuccessful. The same researchers conducted a follow-up investigation to examine the longitudinal molecular pathways involved in milk synthesis/secretion in a model group supplemented with fenugreek [50]. The data imply that fenugreek prolongs peak milk production during mid-lactation through insulin secretion stimulation and regulation of the insulin/GH/IGF-1 axis. The data also implied that fenugreek increases milk ejection by activation of the oxytocin secretion.

### 4.8. Regulation of Fat Metabolism

Obesity is a long-term carbohydrate and lipid metabolic condition marked by excessive fat accumulation in adipose tissue and other internal organs. Insulin resistance, type 2 diabetes, coronary heart disease, cancer, and respiratory disorders are all linked to obesity [51]. Many drugs have been used to treat obesity over the years, but the majority of them have now been removed due to harmful side effects. Fenugreek has been demonstrated in several trials to have anti-obesity characteristics, making it a promising plant option for treating obesity. Fenugreek has a great deal of soluble fibre, which helps to speed up weight reduction by improving digestion and metabolism. Galactomannan, a water-soluble fibre contained in fenugreek seeds, suppresses hunger by increasing the sense of fullness, which aids weight loss. Overall, it enhances glucose and lipid metabolism, insulin sensitivity, antioxidant defence, and lipogenic enzyme downregulation [52]. In vivo, diosgenin may also decrease cholesterol production. The mechanisms of saponins in dyslipidemia-ameliorating actions were initially explained by accelerating cholesterol metabolism and reversing cholesterol transport, as well as blocking 3-hydroxy-3-methylglutaryl coenzyme A reductase in serum and liver [53].

### 4.9. Neuroprotective Effect

Neurological illnesses, such as neuropathic pain, are among the most common, and empirical data show that inflammatory cytokines and microglial cells play a role in the aetiology of neuropathic pain [54]. Using animal models, researchers have revealed the potential benefits of medicinal herbs for the treatment of neurological illnesses. Fenugreek has also been investigated as a potential medicinal herb for the treatment of neurological illnesses in this respect. Khalil et al. [55] fed rats fenugreek saponins (0.05–2.0%) for 45 days and discovered that the dietary treatment of fenugreek-saponins-inhibited apoptosis and acetylcholinesterase (AChE) activity resulted in neuroprotective benefits. Similarly, Bin-Hafeez et al. [29] used a mouse model to study the neuroprotective effects of 5% fenugreek seed powder on aluminium-chloride-induced neurotoxicity and found that fenugreek seed powder had a substantial neuroprotective impact. Trigonella (100 mg/kg) has also been shown to play a function in lowering the risk of Parkinson’s disease by avoiding rotational behaviour and restoring SNC (substantia nigra compact) neuron and MDA (malondialdehyde) levels [56]. Table 4 encapsulates the pharmaceutical properties of fenugreek and its effects on various test models, which have been discussed.

## 5. Conclusions

Herbs are utilised medicinally in a variety of countries owing to the widespread idea that natural items have no side effects and are readily available. In this overview, the physical and chemical properties of fenugreek, as well as traditional usage and pharmacological effects discovered in diverse investigations, are described. These include in-vitro, in vivo, and clinical studies for the past few decades. It is shown that the fenugreek is not only a dietary supplement but also houses potential drug compounds for the treatment of various health conditions. However, studies regarding the mechanism of action of fenugreek and the associated signalling pathways that are specific to a particular disease are crucial in expanding the full potential of fenugreek. Knowing this information will enable researchers to pinpoint compounds and their targets, which, eventually, can be translated into drug development. Because fenugreek grows in a variety of climates around the world, it may have a large range of genotypes. An extensive genomic characterization could further help in identifying the genes responsible for its medicinal effect. As a result, more high-quality research is needed to fully demonstrate the fenugreek plant’s clinical usefulness. Once this barrier is overcome, it can be properly acknowledged as a good plant candidate with a high prospect of being used as a credible medicinal plant to derive new drugs.

## Figures and Tables

**Table 1:** Vitamin profiles and their respective concentrations in fenugreek [5].

Particular	Plant part	Units	Value/100 g
Ascorbic acid	Seed	mg	12–23
Ascorbic acid	Leaves	mg	52.0
Pyridoxine	Seed	mg	0.60
Retinol	Seed	IU	60–100
Niacin	Seed	mg	6.0
β-carotene	Seed	µg	96
β-carotene	Leaves	mg	2.3
Thiamine	Seed	µg	340
Thiamine	Leaves	µg	40
Riboflavin	Seed	µg	290
Riboflavin	Leaves	µg	310
Folic acid	Seed	µg	84

**Table 2:** Chemical constituents of fenugreek seed [8].

	Chemical Constituents of Fenugreek Seed
Alkaloids	trimethylamine, neurin, trigonelline, choline, gentianine, carpaine betain
Amino acids	isoleucine, 4-hydroxyisoleucine, histidine, leucine, lysine, L-tryptophan, argenine
Saponins	graecunins, fenugrin B, fenugreekine, trigofoenosides A-G
Steroidal sapinogens	yamogenin, diosgenin, smilagenin, sarsasapogenin, tigogenin, neotigogenin, gitogenin, yuccagenin, saponaretin
Flavonoids	quercetin, rutin, vitexin, isovitexin
Fibres	gum, neutral detergent fibre
Lipids	triacylglycerols, diacylglycerols, monoacylglycerols, phosphatidylcholine, phosphatidylethanoamine, free fatty acids
Others	coumarin, lipids, vitamins, minerals. 28% mucilage; 22% proteins; 5% of a stronger-swelling, bitter fixed oil

**Table 3:** Traditional medicinal uses of fenugreek.

Traditional Uses	Reference
Demulcent, lactation stimulant, and laxatives	[14]
Aid labour, period cramps, and tonic for metabolism	[15]
Increase milk production in breastfeeding mothers and relieve menstrual cramps, treat cellulitis, boils, and tuberculosis	[15]
Dysmenorrhoeal and postmenopausal symptoms	[6]
Topical effect in soothing irritation caused by eczema	[16]
Lower the amount of calcium oxalate, which is a crystal that causes the formation of kidney stones	[16]
Detoxifying agent in removing toxic wastes, dead cells, and trapped protein through the lymphatic system	[6]

**Table 4:** Pharmaceutical properties of fenugreek.

Pharmaceutical Properties	Plant Part	Effects	Model	Reference
Hypoglycaemic	Seed	Fenugreek improves peripheral glucose utilization and tolerance	Non-insulin-dependent diabetic patients	[4]
		Improvement in glycaemic control among patients with mild type 2 diabetes mellitus	Patients with type II diabetes	[18]
		Dialyzed fenugreek seed extract was comparable to that of insulin	Alloxan-induced diabetic mice	[19]
		Improves glucose control as well as decreasing insulin resistance	Double-blind placebo study	[20]
		Fenugreek seed polyphenols improved insulin signalling and sensitivity compared to metformin-treated rats	Fructose-fed rats	[21]
		Reduction in serum glucose and an increase in liver glycogen	Type 2 diabetic rat	[22]
		Concurrent administration of fenugreek increased the bioavailability of metformin	Rat animal model	[23]
Hypocholesterolemic	Seed	Reduction in total cholesterol and low-density lipoprotein (LDL)	Hypercholesterolemia patients	[8]
		Lower blood lipids, total cholesterol, and triglycerides without affecting the high-density lipoprotein	Patients with coronary heart disease	[8]
		18 to 20% reduction in plasma and liver cholesterol	Ethanolic fenugreek seed-extract-fed rats	[25]
		Lower LDL, total cholesterol, and triglycerides	Fenugreek-seed-powder-treated newly diagnosed type II diabetes patients	[27]
Immunomodulatory	Seed	Stimulatory effect on the body and organ weight, haemagglutinin titre, quantitative haemolysis assay, late-type hypersensitivity response, plaque-forming assay, phagocytic activity, and capacity of macrophages	Swiss albino mice treated with aqueous fenugreek extract	[29]
		Stimulation of the humoral immunity and has anti-inflammatory properties	Mice treated with ethanolic fenugreek extract	[30]
		Regulates the expression of pro-inflammatory marker and immunoregulator marker M1 and M2, respectively	THP-1 macrophages	[31]
		Elevation of the CD4+ and CD8+ values	Streptozotocin-induced diabetic rats	[32]
Antimicrobial	Seed, Leaves and Stem	Methanolic extract had antibacterial affect, but the aqueous extract did not show any activity. The magnitude of effects differs with the plant parts and species of microorganism, as well as the extraction solvent used	Well diffusion involving *E. coli*, *P. aeruginosa*, and *B. cereus*, and various fungal strains	[26,34]
Anticancer	Seed	Potent cytotoxic effect of whole extract compared to purified compound	Prostate cancer cell lines, breast cancer cell lines, and pancreatic cancer cell lines	[35]
		Selective cytotoxicity effect of fenugreek extract	T cell lymphoma	[36]
		Alcoholic fenugreek extract showed in vitro cytotoxicity	IMR-32, a neuroblastoma cell line, and HT29, a cancer cell line	[37]
		Decrease in cell viability and early apoptotic changes	MCF-7 cells, a breast cancer cell line	[38]
		Anti-metastatic effect, induced the inhibition of cell migration and increase in late apoptosis, upregulation of p53	MCF-7 and SK-BR3 breast cancer cell lines	[39]
		IC50 at 25 μg/mL, better pancreatic tissue, higher survival rate	BXPC-3 pancreatic cancer cell line and albino mice	[40]
Antioxidative	Seed	Radical scavenging activity	Biochemical assay	[41]
		Protective effects on lipid peroxidation and enzymatic antioxidant	Cyclophosphamide-treated mice	[42]
		Highest superoxide and free radical scavenging due to high phenolic compound	NBT assay and H_2_O_2_ scavenging	[43]
		Positive effect in the regulation of hepatic enzymes	12-month-old mice	[44]
		Increase in antioxidant radical scavenging activity	DPPH and ABTS assays	[11]
Hormonal effects	Seed	Larger pain reduction and duration of pain decreased	Double-blind, randomized, placebo-controlled trial	[45]
		Reduction in lower abdominal pain	Patients with primary dysmenorrhea	[47]
		Improvement in bone structure and strength	Ovariectomised Wistar rats	[48]
		Increase in milk production	Pregnant Sprague–Dawley rats	[49]
		Modulation of the insulin/GH/IGF-1 axis, stimulation by insulin, and oxytocin secretion	Pregnant Sprague–Dawley rats	[50]
Fat metabolism	Seed	Helps to speed up weight reduction by improving digestion and metabolism	Fat-induced obese rat	[52]
		Suppresses hunger by increasing the sense of fullness, which aids weight loss	Fat-induced obese rat	[52]
		Accelerating cholesterol metabolism and reversing cholesterol transport, as well as blocking 3-hydroxy-3-methylglutaryl coenzyme A reductase in serum and liver	In vivo	[53]
Neuroprotective effects	Seed	Fenugreek-saponins-inhibited apoptosis and acetylcholinesterase (AChE) activity	Rats	[55]
		Substantial neuroprotective impact	Aluminium-chloride-induced neurotoxicity mouse	[29]
		Avoiding rotational behaviour and restoring SNC (substantia nigra compact) neuron and MDA (malondialdehyde) levels	Trigonella-fed mouse	[56]

## Data Availability

Not applicable.
